# Supplementary material for: Antiaging Properties of Kalanchoe blossfeldiana Ethanol Extract—Ex Vivo and In Vitro Studies
Source: Molecules. 2024 Nov 24;29(23):5548. doi: 10.3390/molecules29235548 (PMC11643982; doi:10.3390/molecules29235548)
Supplement: Supplementary file 1 [file molecules-29-05548-s001.zip › Figure S1 Supplementary Materials.pdf]

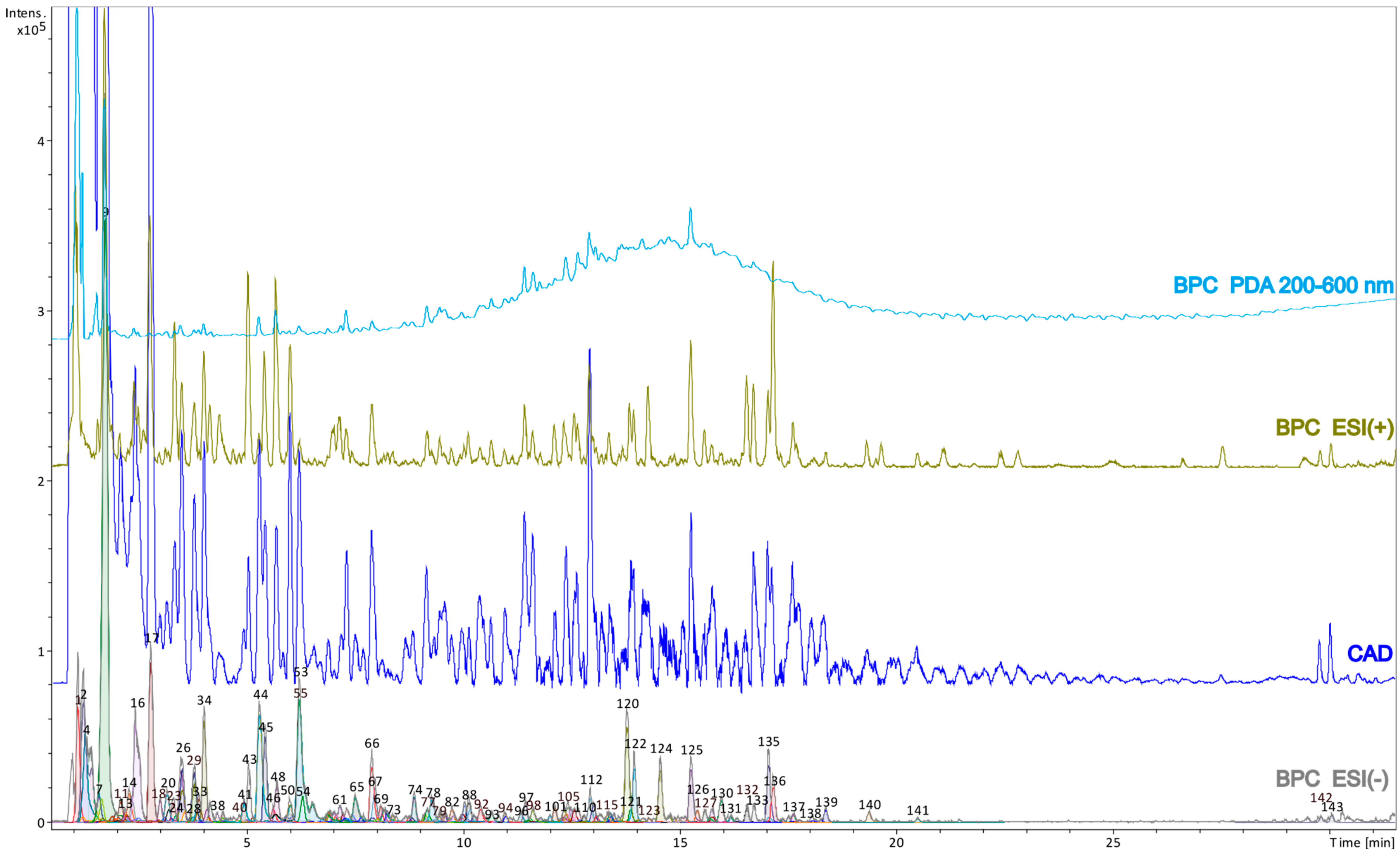


**Figure S1.** Base peak chromatograms (ESI(-), ESI(+), PDA) and CAD chromatogram from the UHPLC-HR-MS analyses of *Kalanchoe blossfeldiana* extract. Peak numbers as in Table S1.
